# Excitable Neurons, Firing Threshold Manifolds and Canards

**DOI:** 10.1186/2190-8567-3-12

**Published:** 2013-08-14

**Authors:** John Mitry, Michelle McCarthy, Nancy Kopell, Martin Wechselberger

**Affiliations:** 1 School of Mathematics and Statistics, University of Sydney, Sydney, NSW, Australia; 2 Department of Mathematics, Boston University, Boston, MA, USA

## Abstract

We investigate firing threshold manifolds in a mathematical model of an excitable neuron. The model analyzed investigates the phenomenon of post-inhibitory rebound spiking due to propofol anesthesia and is adapted from McCarthy et al. (SIAM J. Appl. Dyn. Syst. 11(4):1674–1697, [2012]). Propofol modulates the decay time-scale of an inhibitory GABAa synaptic current. Interestingly, this system gives rise to rebound spiking within a specific range of propofol doses. Using techniques from geometric singular perturbation theory, we identify geometric structures, known as canards of folded saddle-type, which form the firing threshold manifolds. We find that the position and orientation of the canard separatrix is propofol dependent. Thus, the speeds of relevant slow synaptic processes are encoded within this geometric structure. We show that this behavior cannot be understood using a static, inhibitory current step protocol, which can provide a single threshold for rebound spiking but cannot explain the observed cessation of spiking for higher propofol doses. We then compare the analyses of dynamic and static synaptic inhibition, showing how the firing threshold manifolds of each relate, and why a current step approach is unable to fully capture the behavior of this model.

## 1 Introduction

Excitable neurons are typically at rest, but can fire action potentials in response to certain forms of stimulation. Many textbooks refer to excitability as an all-or-none response, i.e. a “subthreshold” synaptic input leads to a small graded postsynaptic potential while a “superthreshold” input evokes an action potential. One then seeks to find an action potential threshold, i.e., a particular voltage value that demarcates the all-or-none response. However, this is a misconception. Using geometric analysis of neural models, it was FitzHugh [10] who first noticed that a firing threshold, if it exists, is never a number but a manifold. 

Another characteristic feature of neurons is the existence of processes that evolve on multiple time-scales. The interaction of ionic currents acting on different time-scales is responsible for the creation of action potentials in neurons. This time-scale feature leads to mathematical models of neurons often referred to as singular perturbation problems. These models are particularly amenable to analysis using geometric singular perturbation theory (GSPT) [9,14] with the specific aim of giving predictions of model dynamics based on singular limit observations. Threshold phenomena are closely related to folded critical manifolds of such singularly perturbed neural problems. In the famous 2-dimensional singularly perturbed FitzHugh–Nagumo model, it is the repelling middle branch of the folded (cubic-shaped) critical manifold that forms the firing threshold manifold in the singular limit. In the full system, this firing threshold manifold perturbs to a nearby repelling slow manifold. 

In higher dimensional models with more than one slow variable, the geometry of such singularly perturbed problems becomes quite intricate and folded singularities play a prominent role. Folded singularities lie on a fold of the critical manifold where stable and unstable branches of this (higher dimensional) manifold meet. *Canards*[2,3,8,15,21,23,24] are trajectories associated with folded singularities and connect the stable and unstable branches of the critical manifold. These special solutions have been identified as important objects in explaining complex oscillatory patterns known as mixed-mode oscillations (MMOs) [4]. There now exists a substantial amount of literature on applications of canard theory, and we refer the interested reader to detailed tables on relevant literature provided in a review on MMOs [5]. 

Canards form boundaries of different dynamic behavior—they are separatrices by nature. More importantly, they encode slow time-scales, i.e., temporal information is reflected in the geometry of a canard. In the two-dimensional case, Izhikevich [13] clearly highlights the fact that canard trajectories and repelling slow manifolds provide the best approximation to the firing threshold manifold, hence giving a mathematical “structure” to the famous *No Man’s Land* by R. FitzHugh [11]. More recently, Desroches et al. [6] discuss the relationship between canards and excitability thresholds in planar slow-fast systems by identifying inflection lines of the flow. 

In the present study, we focus on a higher dimensional neural model and the specific role of folded saddle canards as firing threshold manifolds. We observe that varying one of the slow time-scales changes the boundaries of different dynamic behaviors. The slow time-scale is thus encoded in the position of the saddle canard separatrix, and so, remarkably, a change in a slow time-scale can be “seen” in a geometric object. Folded saddle canards have been shown to form firing threshold manifolds in general Morris–Lecar/FitzHugh–Nagumo type neural models with dynamic current input [25] and even in a climate model of the so-called “compost-bomb instability” by Wieczorek et al. [26]. In both, under variation of a particular parameter, the position of a folded saddle canard varies explaining the excitability properties of the model. 

This geometric observation becomes important when we try to understand changes in neural dynamics. Neurons constantly sense their environment, e.g., temperature, acidity or glucose, and they encode fluctuations of these parameters by changing their ion channel dynamics. The famous Hodgkin–Huxley model of the squid giant axon [12] incorporates temperature changes through a $Q_{10}$-temperature factor that increases the speed of the ion channel gates with increasing temperature. If such environmental changes are encoded in different speeds of slow ion channels, then we expect this to be reflected in a change of the firing threshold manifold of the neuron. Thus, identifying the cause of different neural dynamics through the specific position of a canard in a singularly perturbed system becomes a valuable diagnostic tool to understand this phenomenon, and it is the focus of this work.

## 2 Propofol and Rebound Spiking

As a case study, we investigate the role of the general anesthetic propofol on rebound spiking in the central nervous system [17]. Many general anesthetics, including propofol, prolong the duration of GABAergic inhibitory postsynaptic currents (IPSCs), and this action contributes to the behavioral properties of these drugs [1]. Mathematically speaking, propofol changes the slow time-scale of the deactivation of the GABAa receptor channel. Paradoxically, low doses of propofol causes excitation rather than sedation. This behavior can already be observed in an isolated single cell model that receives GABAergic IPSCs [18]. We adapt this propofol neuron model formulated in [18] slightly in order to more clearly emphasize the role of folded saddle canards in the observed dynamic behavior. We note that only minimal adjustments have been made in order to preserve the qualitative behavior of the model, namely the observation of post-inhibitory rebound spiking for a window of GABAa synaptic time-scale values. The modification consists of two parameter changes, the details of which are given below. The essential difference is that this modification shifts the resting membrane potential to a lower, more hyperpolarized, voltage value, allowing a more uniform separation of time-scales over a range of GABAa time-scale values. This modification enables us to make full use of the machinery of GSPT. More details of the relation between the two models can be found in the last section of the paper. 

The modified propofol model is given as a six-dimensional conductance based model with Hodgkin–Huxley type dynamics:

(1) $$\begin{array}{rl} c_{m}\frac{dV}{dt} & =-I_{\mathrm{Na}}-I_{K}-I_{L}-I_{M}-I_{\mathrm{syn}}+I_{\mathrm{app}},\\ \frac{dx}{dt} & =\frac{\left(x_{\infty }(V)-x\right)}{\tau_{x}(V)},\;x=m,h,n,w,\\ \frac{ds}{dt} & =-s/\tau_{s}, \end{array}$$

where the ionic currents are defined as

(2) $$\begin{array}{rl} I_{\mathrm{Na}} & =g_{\mathrm{Na}}m^{3}h(V-E_{\mathrm{Na}}),\\ I_{K} & =g_{k}n^{4}(V-E_{k}),\\ I_{L} & =g_{L}(V-E_{L}),\\ I_{M} & =g_{m}w(V-E_{k}),\\ I_{\mathrm{syn}} & =g_{i}s(V-E_{i}). \end{array}$$

The membrane currents consist of a fast sodium current, $I_{\mathrm{Na}}$, a potassium current, $I_{K}$, and a leak current, $I_{L}$, collectively referred to as the spiking currents, and a slow muscarinic potassium M-current, $I_{M}$. This model neuron receives GABAa IPSCs, here modeled by the current $I_{\mathrm{syn}}$. The applied current, $I_{\mathrm{app}}$, models tonic external drive.

**Fig. 1:**
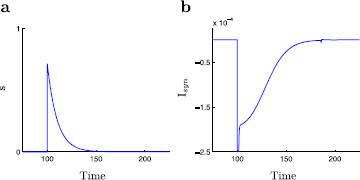
The inhibitory gating variable, *s* and the inhibitory current, $I_{\mathrm{syn}}$. The dimensionless modified propofol model is simulated with inhibition modeled at t=100. Here $\tau_{s}=10$. **a** A time-trace of *s*. The dynamics of the gating variable *s* are decoupled from the rest of the system. The gating variable decays exponentially with decay constant $\tau_{s}$ where the value of this decay constant is linked to the amount of propofol present. **b** A time-trace of $I_{\mathrm{syn}}$. Recall the synaptic current is given by $I_{\mathrm{syn}}=g_{i}s(V-E_{i})$. At the onset of dynamic behavior the synaptic current exhibits a rapid spike followed by an approximately exponential decay. Note the small kink at approximately t=180 due to the spike (action potential) in *v*

As is typical in Hodgkin–Huxley type conductance based models, gating activation and deactivation variables model changes in ion channel conductances. Each take values restricted within the interval [0,1] reflecting opening probabilities. Here, *m*, *n*, and *w* are gating activation variables, whereas *h* is a gating deactivation variable. Similarly, the variable *s* is a gating variable for synaptic GABAa receptor channels. The value of *s* corresponds to the inhibitory input the neuron receives. In order to model inhibitory postsynaptic input in the absence of a network of neurons, the variable *s* is set to $s_{0}=0.714$ at the instantaneous activation of the GABAa receptor channel. This *s* value is used as it most closely reflects the dynamic change in synaptic input within a network of coupled neurons. The deactivation of this channel is then simply modeled by (slow) exponential decay with time constant $\tau_{s}$; see Fig. 1 for a comparison of the synaptic gating variable *s* and the synaptic inhibitory current $I_{\mathrm{syn}}$. Since propofol alters the deactivation time-scale of GABAa receptor channels, we consider $\tau_{s}$ as our main study parameter. 

The explicit forms of the steady state functions, $x_{\infty }(V)$, and the time-scale functions, $\tau_{x}(V)$, are given in the Appendix (see Fig. 15 for plots). The model constants are given in Table 1. Note the two modifications from the original propofol model in [18]: $g_{i}=4\text{ mS}$ (originally $g_{i}=0.04\text{ mS}$) and the *w*-nullcline is shifted by 3 mV in the direction of negative *v*. 

**Table 1:** Propofol neuron and network model system parameters

Parameter	Value	Description
*Current balance equation constants*		
$c_{m}$	1 μF	Membrane capacitance
$I_{\mathrm{app}}$	1.81 μA	Applied current
*Maximal ion channel conductances*		
$g_{\mathrm{Na}}$	100 mS	Voltage-gated Na^+^ channels
$g_{k}$	80 mS	Voltage-gated K^+^ channels
$g_{L}$	0.1 mS	Leak channels
$g_{m}$	2 mS	Slow acting voltage-gated K^+^ channels
$g_{i}$	4 mS	Synaptic GABAa receptor channels
*Ionic current reversal potentials*		
$E_{\mathrm{Na}}$	50 mV	Voltage-gated Na^+^ channels
$E_{k}$	−100 mV	Voltage-gated K^+^ channels
$E_{L}$	−67 mV	Leak channels
$E_{i}$	−80 mV	Synaptic GABAa receptor channels

### 2.1 Dynamic Inhibition, but not the Current Step Protocol, Leads to Cessation of Spiking with Increased Inhibition

**Fig. 2:**
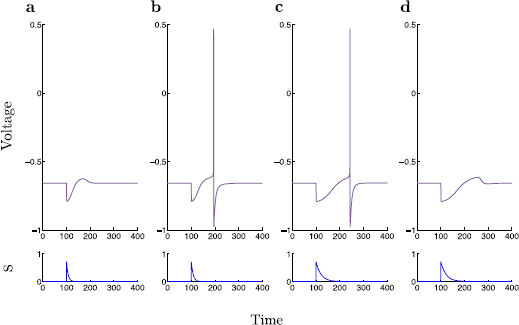
Rebound spiking with dynamic inhibition. The dimensionless modified propofol model is simulated for four different values of $\tau_{s}$, 7, 8, 21, and 22. **a**$\tau_{s}=7$, **b**$\tau_{s}=8$, **c**$\tau_{s}=21$, **d**$\tau_{s}=22$. Voltage time-traces are shown within each panel, and the corresponding time-traces of *s* are shown below. Note that there exists a range of $\tau_{s}$ values for which rebound spiking occurs, specifically for $\tau_{s}\in [8,21]$

**Fig. 3:**
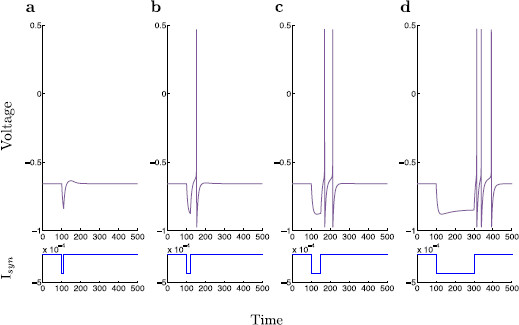
Rebound spiking with step inhibition. A classical current step protocol is applied to the dimensionless modified propofol model. Here, the value of $I_{\mathrm{syn}}$ is held constant at $3.5\times 10^{-4}$, roughly mimicking the slowly decaying portion of the dynamic inhibition current. The protocol is simulated for four different durations, 10, 20, 50, and 200 ms. **a** Duration = 10 ms, **b** Duration = 20 ms, **c** Duration = 50 ms, **d** Duration = 200 ms. Voltage time-traces are shown within each panel, and the corresponding time-traces of $-I_{\mathrm{syn}}$ are shown below. As opposed to dynamic inhibition, here we observe no cessation of spiking for prolonged inhibitory input. On the contrary, we observe an isolated couplet of spikes for prolonged inhibition, and as the duration of the inhibitory step is further increased, we observe a triplet of spikes

Classically, an applied current step protocol has been used to understand rebound spiking [13,19]. However, as was pointed out in [18], we find here that it is necessary to consider the dynamic interplay between the inhibitory synaptic current and the slow potassium M-current in order to explain the observed behavior of spiking within the modified propofol model (compare Figs. 2 and 3). Within the modified propofol model, there exists a specific range of values of $\tau_{s}$ for which the system exhibits spiking behavior. Outside this range, the system fails to spike (Fig. 2). This matches well with the experimental observation that only low doses of propofol are able to create the state of paradoxical excitation [20]. 

Using a traditional approach, the spiking behavior is studied with a step protocol. Here, an applied current is switched on, kept at a constant level, then removed. By holding $I_{\mathrm{syn}}$ constant, the dynamics in *s* are lost, thus rendering the step protocol system five-dimensional as opposed to the six-dimensional dynamic inhibition system (1). This step protocol is usually able to reproduce spiking patterns, while simplifying input dynamics, and thus give insight into the associated spiking behavior. However, applying a step protocol to the present model, we find a single transition from inactivity to isolated spiking as $\tau_{s}$ is increased. As $\tau_{s}$ is further increased a transition from single spiking to a doublet, and then to a triplet of spikes is observed (Fig. 3). A maximum of three spikes is observed despite further increases in duration of inhibition. The step protocol is unable to reproduce a cessation of spiking for increased synaptic inhibition. It thus becomes apparent that there is a necessary dynamic mechanism required to yield a specific range of spiking under variation of synaptic inhibition time-scale.

In the present study, our aim is to identify firing threshold manifolds of the propofol model, both with dynamic and with static inhibition, thus explaining the spiking behavior under variation of propofol dosage and duration of current step inhibition, respectively. Key to this aim is the use of GSPT for which a detailed analysis of time-scales is necessary. This time-scale analysis is presented in Sect. 3. Identifying multiple time-scales in system (1) implies a splitting of solution trajectories of (1) into segments of fast and slow dynamics. These fast and slow dynamics are captured by lower dimensional subsystems, termed the layer and reduced problems, respectively. GSPT uses these lower dimensional subsystems, studied in Sect. 4, to provide insight into the geometric structures which govern the behavior of the model, and thus to predict the dynamics of the full (higher dimensional) system (1). Results are given in Sect. 5 for the case of dynamic inhibition. In particular, we identify a singular canard of folded saddle type as the separatrix that forms the firing threshold manifold. In Sect. 6, a similar analysis is carried out for the case of static inhibition; again a singular limit prediction of the spiking threshold manifold is identified. In both the dynamic and static inhibition cases, a numerical confirmation of singular limit predictions is made by calculating the true firing threshold manifolds. This emphasizes the predictive power of GSPT. We also point out that the firing threshold manifold in the case of static inhibition is in fact the structure which the firing threshold manifold in the case of dynamic inhibition approaches in the limit as $\tau_{s}\to 0$, i.e., in the transition from slow to fast synaptic GABAa inhibition decay rates. In Sect. 7, we discuss the original and modified propofol models and their respective analyses. Finally, in Sect. 8, we make some concluding remarks about canards and excitability in neural models.

## 3 Time-Scales and Dimensional Analysis

By observation of the time traces in Figs. 2 and 3, it can be argued that there exists a multiple time-scales structure within the typical solution trajectories of the modified propofol model (1). In order to investigate this behavior further, a dimensional analysis of the system is performed so as to roughly determine the time-scales on which each variable evolves.

### 3.1 The Modified Propofol Model Has Three Distinct Time-Scales

As part of the nondimensionalization procedure, we introduce the three scaling constants, $k_{v}$, $k_{t}$, and $g_{\max}$. The membrane voltage is scaled according to $V=vk_{v}$, where $k_{v}$ is the typical range over which the modified propofol model trajectories evolve in the *V*-direction. Time is scaled as $t=\tau k_{t}$, where $k_{t}$ has units of *ms* and is determined below. The ion channel conductances are scaled according to $g_{x}={\bar{g}}_{x}g_{\max}$, where $g_{\max}=\max\{g_{\mathrm{Na}},g_{k},g_{L},g_{m},g_{i}\}$. This leads to a dimensionless form of the modified propofol model (1),

(3) $$\begin{array}{rl} \frac{c_{m}}{k_{t}g_{\max}}\frac{dv}{d\tau } & =-{\bar{I}}_{\mathrm{Na}}-{\bar{I}}_{K}-{\bar{I}}_{L}-{\bar{I}}_{M}-{\bar{I}}_{\mathrm{syn}}+{\bar{I}}_{\mathrm{app}},\\ \frac{\tau_{x}}{k_{t}}\frac{dx}{d\tau } & =\frac{\left({\tilde{x}}_{\infty }(v)-x\right)}{{\tilde{\tau }}_{x}(v)},\;x=m,h,n,w,\\ \frac{\tau_{s}}{k_{t}}\frac{ds}{d\tau } & =-s, \end{array}$$

where the ionic currents are now defined as

(4) $$\begin{array}{rl} {\bar{I}}_{\mathrm{Na}} & ={\bar{g}}_{\mathrm{Na}}m^{3}h(v-e_{\mathrm{Na}}),\\ {\bar{I}}_{K} & ={\bar{g}}_{k}n^{4}(v-e_{k}),\\ {\bar{I}}_{L} & ={\bar{g}}_{L}(v-e_{L}),\\ {\bar{I}}_{M} & ={\bar{g}}_{m}w(v-e_{k}),\\ {\bar{I}}_{\mathrm{syn}} & ={\bar{g}}_{i}s(v-e_{i}). \end{array}$$

The dimensionless steady state functions and time-scale functions are given as ${\tilde{x}}_{\infty }(v)=x_{\infty }(vk_{v})$ and ${\tilde{\tau }}_{x}(v)=\tau_{x}(vk_{v})/\tau_{x}$, respectively. Note that the function $\tau_{x}(vk_{v})$ has been scaled by

$$\tau_{x}=\min_{e_{K}\leq v\leq e_{\mathrm{Na}}}\tau_{x}(vk_{v})$$

in order to yield the dimensionless time-scale function ${\tilde{\tau }}_{x}(v)$. Hence, the right-hand sides in system (3) are all of order O(1) and we can identify roughly the typical time-scales on the left-hand side:

(5) $$\begin{array}{ll} \tau_{v}:=c_{m}/g_{\max}=0.01\;\mathrm{ms}, & \tau_{m}=0.03\;\mathrm{ms}\;(0.05\;\mathrm{ms}),\\ \tau_{h}=0.25\;\mathrm{ms}\;(0.5\;\mathrm{ms}), & \tau_{n}=0.3\;\mathrm{ms}\;(0.7\;\mathrm{ms}),\\ \tau_{w}=38\;\mathrm{ms}\;(46\;\mathrm{ms}), & \tau_{s}\in [3,100]\;\mathrm{ms}. \end{array}$$

Note that the $\tau_{x}$, x=m,h,n,w values given in parentheses refer to the time-scale functions for subthreshold v≤−0.5. The time-scale on which *s* evolves is given directly by the value of $\tau_{s}$.

It is now possible, based on the above numbers, to propose a hierarchy of variables according to the time-scales on which they evolve. We classify the variables *v* and *m* superfast, *h* and *n* fast, and *w* and *s* slow. Recall the value of $\tau_{s}$ directly determines the time-scale on which *s* evolves. Accordingly, care should be taken in the following singular perturbation analysis since it may no longer be valid to consider *s* slow as $\tau_{s}$ becomes sufficiently small. (In [18], *s* is considered a fast variable when $\tau_{s}$ is small.)

Another way to check time-scales separation is to consider the maximum magnitude of the time derivative of each state variable over the course of a full spiking trajectory; see Fig. 16. For $\tau_{s}=8$, we observe that the $\max\{|\frac{dx}{dt}|\}$ for x=v,m,h,n,w,s is roughly given by (10.2,6.4,3.3,2.1,0.025,0.089); compare these values with the inverse of the time-scales given in (5). This suggests that our proposed time-scale hierarchy with *w* and *s* slow is reasonable. As alluded to earlier, the specific insight of this model is the interplay between the slow M-current and the inhibitory synaptic current contributing to rebound spiking.

We set $k_{v}=100\text{ mV}$ as a typical voltage scale for a spiking neuron and $k_{t}=1\text{ ms}$ as a typical fast time-scale of the “fast” variables (n,h). Thus, system (3) becomes

(6) $$\begin{array}{rl} \delta \frac{dv}{d\tau } & =-{\bar{I}}_{\mathrm{Na}}-{\bar{I}}_{K}-{\bar{I}}_{L}-{\bar{I}}_{M}-{\bar{I}}_{\mathrm{syn}}+{\bar{I}}_{\mathrm{app}}=:f_{1}(v,m,h,n,w,s),\\ \delta \frac{dm}{d\tau } & =\frac{c_{m}}{\tau_{m}g_{\max}}\cdot \frac{\left({\tilde{m}}_{\infty }(v)-m\right)}{{\tilde{\tau }}_{m}(v)}=:f_{2}(v,m),\\ \frac{dh}{d\tau } & =\frac{k_{t}}{\tau_{h}}\cdot \frac{\left({\tilde{h}}_{\infty }(v)-h\right)}{{\tilde{\tau }}_{h}(v)}=:f_{3}(v,h),\\ \frac{dn}{d\tau } & =\frac{k_{t}}{\tau_{n}}\cdot \frac{\left({\tilde{n}}_{\infty }(v)-n\right)}{{\tilde{\tau }}_{n}(v)}=:f_{4}(v,n),\\ \frac{dw}{d\tau } & =\varepsilon \frac{\left({\tilde{w}}_{\infty }(v)-w\right)}{{\tilde{\tau }}_{w}(v)}=:\varepsilon g_{1}(v,w),\\ \frac{ds}{d\tau } & =\varepsilon \left(-\frac{\tau_{w}}{\tau_{s}}s\right)=:\varepsilon g_{2}(s), \end{array}$$

where $\delta :=c_{m}/(k_{t}g_{\max})=0.01\ll 1$ measures the time-scale separation between the fast variables (h,n) and the superfast variables (v,m) while $\varepsilon :=k_{t}/\tau_{w}=0.025\ll 1$ measures the time-scale separation between the fast variables (h,n) and the slow variables (w,s). Thus, the dimensionless system (6) is a singularly perturbed problem with singular perturbation parameters δ≪1 and ε≪1. This suggests an inherent three time-scales problem. The specific insight of the model and, therefore, the corresponding analysis should focus on the interplay of the slow M-current and the inhibitory synaptic current contributing to rebound spiking. We therefore group the fast and superfast variables together into one “fast” pool and consider system (6) a 4-fast/2-slow (v,m,h,n)/(w,s) problem. In doing so, we choose *ε* as the main singular perturbation parameter and keep *δ* fixed.

## 4 Geometric Singular Perturbation Theory

The identification of distinct fast and slow time-scales in the modified propofol model allows us to utilize the methods of GSPT to identify the key differences in geometric structure that account for the differences in spiking dynamics between static and dynamic inhibition. Thus, we proceed to identify two lower dimensional subsystems that govern the fast and slow dynamics in order to give us insight into the behavior of the full higher dimensional system.

### 4.1 Layer Problem

In the limit as ε→0 of system (6), we derive the layer problem, which approximates the fast components (v,m,h,n) of the full system dynamics:

(7) $$\begin{array}{rl} \delta \dot{v} & =f_{1}(v,m,h,n,w,s),\\ \delta \dot{m} & =f_{2}(v,m),\\ \dot{h} & =f_{3}(v,h),\\ \dot{n} & =f_{4}(v,n),\\ \dot{w} & =0,\\ \dot{s} & =0, \end{array}$$

where $\dot{x}$ denotes the derivative of *x* with respect to the fast time-scale, $t_{\mathrm{fast}}=\tau$. Note that the slow variables (w,s) are considered parameters in the layer problem. An important geometric object is the critical manifold, which is defined as the set of equilibria of the layer problem:

(8) $$\begin{array}{rl} S_{0} & =\left\{(v,m,h,n,w,s)\in R^{6}|f_{1}=f_{2}=f_{3}=f_{4}=0\right\}\\ & =\left\{(v,m,h,n,w,s)\in R^{6}|f_{1}\left(v,{\tilde{m}}_{\infty }(v),{\tilde{h}}_{\infty }(v),{\tilde{n}}_{\infty }(v),w,s\right)=0\right\}. \end{array}$$

**Fig. 4:**
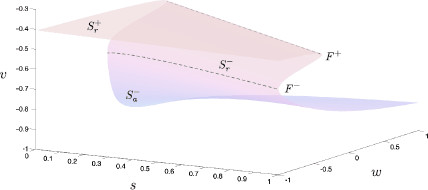
Critical manifold in (v,w,s)-space. The critical manifold, $S_{0}$, is the set of stationary equilibria within the layer problem, (7). This surface defines the interface between fast and slow dynamics: the layer problem describes rapid movement toward or away from $S_{0}$, the reduced problem describes slower dynamics on the manifold itself. Within a physiologically relevant domain, $S_{0}$ has a roughly cubic structure, consists of upper and lower fold curves, $F^{+}$ and $F^{-}$ respectively, and three sheets of stationary equilibria. There exists a single lower attracting sheet, $S_{a}^{-}$, and upper and lower repelling sheets, $S_{r}^{+}$ and $S_{r}^{-}$, respectively. On the uppermost surface lies a line of Andronov–Hopf bifurcations from which a family of limit cycles emanate. The line of Andronov–Hopf bifurcations lies out of range while the family of limit cycles terminate within (not shown). Note the shape and stability properties of the critical manifold are independent of $\tau_{s}$

Note here that $f_{2}=f_{3}=f_{4}=0$ can be solved for $x={\tilde{x}}_{\infty }(v)$, x=m,h,n, yielding a single expression for the critical manifold in (v,w,s)-space. This allows for a projection of the critical manifold into three-dimensional space, shown in Fig. 4. Under variation of *w* and *s*, the two-dimensional critical manifold contains an upper and lower set of fold or saddle-node bifurcations, $F^{+}$ and $F^{-}$, respectively. These fold lines contain equilibria with a single zero eigenvalue and, in effect, partitions $S_{0}$ into three surfaces of equilibria. The lowermost surface of $S_{0}$ is denoted $S_{a}^{-}$ and is an attracting surface of equilibria, i.e., all equilibria have four eigenvalues with negative real parts. The surface bound between the two fold curves, $S_{r}^{-}$, is a repelling surface of equilibria with a single eigenvalue with positive real part. Above the upper fold lies a second repelling surface of equilibria, $S_{r}^{+}$, containing equilibria with two eigenvalues with positive real part. 

Moving further up on the uppermost surface there exists a line of Hopf bifurcations, $H^{+}$, indicating the boundary between $S_{r}^{+}$ and a second attracting surface of equilibria, $S_{a}^{+}$. Stable limit cycles emanate from the Hopf line through supercritical Hopf bifurcations and terminate in homoclinic cycles, which are homoclinic to the lower fold curve, $F^{-}$ (this bifurcation structure is known as a saddle node on invariant cycles, or SNIC, bifurcation). The Hopf line lies outside the physiological range of interest (i.e., w<0); however, the associated limit cycles emanate from outside this region of interest and terminate just within. Thus, the layer problem contains two stable attractors; the lower branch of the critical manifold and the set of stable limit cycles.

### 4.2 Reduced Problem

The reduced problem describes the dynamics of the slow variables (w,s) on the critical manifold. Time is first scaled by *ε* to obtain the following slow system:

(9) $$\begin{array}{rl} \varepsilon \delta v^{\prime } & =f_{1}(v,m,h,n,w,s),\\ \varepsilon \delta m^{\prime } & =f_{2}(v,m),\\ \varepsilon h^{\prime } & =f_{3}(v,h),\\ \varepsilon n^{\prime } & =f_{4}(v,n),\\ w^{\prime } & =g_{1}(v,w),\\ s^{\prime } & =g_{2}(s), \end{array}$$

where $x^{\prime }$ denotes the derivative of *x* with respect to the slow time-scale, $t_{\mathrm{slow}}=\varepsilon \tau$. In the limit as ε→0 of system (9), we derive the reduced problem, which approximates the slow components (w,s) of the full system dynamics

(10) $$\begin{array}{rl} 0 & =f_{1}(v,m,h,n,w,s),\\ 0 & =f_{2}(v,m),\\ 0 & =f_{3}(v,h),\\ 0 & =f_{4}(v,n),\\ w^{\prime } & =g_{1}(v,w),\\ s^{\prime } & =g_{2}(s). \end{array}$$

The reduced problem is a two-dimensional differential algebraic equation. Within (10), the first four equations dictate that the dynamics occur on the critical manifold $S_{0}$, and the last two equations describe the dynamics of the slow variables *w* and *s* thereon. Note the algebraic equation defining $S_{0}$, $f_{1}(v,{\tilde{m}}_{\infty }(v),{\tilde{h}}_{\infty }(v),{\tilde{n}}_{\infty }(v),w,s)=0$, can be solved for w=w(v,s) respectively s=s(v,w), but not for *v* reflecting the folded geometry of the manifold; see Fig. 4. Hence, it suffices to study the flow on the critical manifold $S_{0}$ in one single coordinate chart, either the (v,s)-chart or the (v,w)-chart where $S_{0}$ is defined as a graph.

By definition, the reduced vector field is in the tangent bundle of the critical manifold $S_{0}$. This condition is encoded in the total time derivative of $f_{1}=0$, i.e.,

(11) $$f_{1}^{\prime }\left(v,{\tilde{m}}_{\infty }(v),{\tilde{h}}_{\infty }(v),{\tilde{n}}_{\infty }(v),w,s\right)=\Phi (v,w,s)v^{\prime }+\frac{\partial f_{1}}{\partial w}w^{\prime }+\frac{\partial f_{1}}{\partial s}s^{\prime }=0,$$

where, once again, a prime denotes a derivative with respect to slow time and

(12) $$\Phi (v,w,s):=\frac{\partial f_{1}}{\partial v}+\frac{\partial f_{1}}{\partial m}\frac{d{\tilde{m}}_{\infty }}{dv}+\frac{\partial f_{1}}{\partial h}\frac{d{\tilde{h}}_{\infty }}{dv}+\frac{\partial f_{1}}{\partial n}\frac{d{\tilde{n}}_{\infty }}{dv}.$$

Projecting the reduced problem onto the (v,s)-chart gives

(13) $$\begin{array}{rl} -\Phi \left(v,w(v,s),s\right)v^{\prime } & =\frac{\partial f_{1}}{\partial w}g_{1}\left(v,w(v,s)\right)+\frac{\partial f_{1}}{\partial s}g_{2}(s),\\ s^{\prime } & =g_{2}(s), \end{array}$$

while projecting the reduced problem onto (v,w)-chart gives the equivalent system

(14) $$\begin{array}{rl} -\Phi \left(v,w,s(v,w)\right)v^{\prime } & =\frac{\partial f_{1}}{\partial w}g_{1}(v,w)+\frac{\partial f_{1}}{\partial s}g_{2}\left(s(v,w)\right),\\ w^{\prime } & =g_{1}(v,w). \end{array}$$

Both systems are singular along the fold curves, that is, where Φ(v,w,s)=0. The singularity can be removed by rescaling time by −Φ(v,w,s). This yields the desingularized reduced system

(15) $$\begin{array}{rl} v^{\prime } & =\frac{\partial f_{1}}{\partial w}g_{1}\left(v,w(v,s)\right)+\frac{\partial f_{1}}{\partial s}g_{2}(s),\\ s^{\prime } & =-\Phi \left(v,w(v,s),s\right)g_{2}(s), \end{array}$$

respectively,

(16) $$\begin{array}{rl} v^{\prime } & =\frac{\partial f_{1}}{\partial w}g_{1}(v,w)+\frac{\partial f_{1}}{\partial s}g_{2}\left(s(v,w)\right),\\ w^{\prime } & =-\Phi \left(v,w,s(v,w)\right)g_{1}(v,w). \end{array}$$

The flow described by (15), respectively (16), is equivalent to that of (13), respectively (14), on the attracting surface, $S_{a}^{-}$, and the repelling surface, $S_{r}^{+}$, while reversed on the repelling surface, $S_{r}^{-}$. This is due to the rescaling of time by −Φ(v,w,s), which is positive on $S_{a}^{-}$ and $S_{r}^{+}$ and negative on $S_{r}^{-}$.

#### 4.2.1 Canards Form a Separatrix for Solutions of the Reduced Problem

Here, we aim to use the geometry of the reduced system in order to identify a manifold that separates the trajectories of the reduced problem into two distinct behaviors: those that return to an equilibrium or rest state and those that proceed to the fold curve where the fast dynamics again become important in establishing a spiking solution. Important to the identification of this manifold is finding folded singularities. We find a folded saddle equilibrium; the canard solution of which is the relevant manifold that separates the behavior of the reduced trajectories.

The desingularized system (15), respectively (16), possesses, in general, two types of equilibria: ordinary singularities and folded singularities,

(17) $$\begin{array}{rl} Q_{O} & =\left\{(v,m,h,n,w,s)\in S_{0}\setminus F|g_{1}(v,w)=g_{2}(s)=0\right\},\\ Q_{F} & =\left\{(v,m,h,n,w,s)\in F|\frac{\partial f_{1}}{\partial w}g_{1}(v,w)+\frac{\partial f_{1}}{\partial s}g_{2}(s)=0\right\}. \end{array}$$

Ordinary singularities $\left(g_{1}=g_{2}=0\right)$ are equilibria of the desingularized flow (15), respectively (16), of the reduced flow, (10), and of the original system, (9). Folded singularities, $\left(\Phi =0,\frac{\partial f_{1}}{\partial w}g_{1}+\frac{\partial f_{1}}{\partial s}g_{2}=0\right)$ on the other hand are generally not equilibria of the reduced flow or the original system.

Within (15), respectively (16), we find three stable nodes, ${\mathrm{eq}}_{1}$, ${\mathrm{eq}}_{2}$ and ${\mathrm{eq}}_{3}$, which constitute the set of ordinary singularities, $Q_{O}$. Note that ${\mathrm{eq}}_{1}$ lies on $S_{r}^{+}$, ${\mathrm{eq}}_{2}$ lies on $S_{r}^{-}$ and ${\mathrm{eq}}_{3}$ lies on $S_{a}^{-}$. The position and stability of these equilibria are independent of the value of the parameter $\tau_{s}$.

**Fig. 5:**
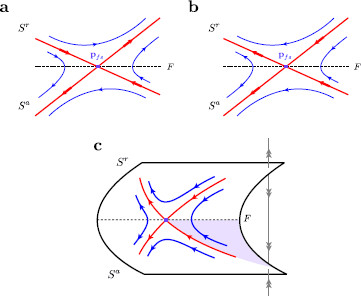
Folded saddle geometry and associated trajectories. The geometry of a generic folded saddle. The folded saddle (*purple*) is denoted $p_{\mathrm{fs}}$, while the fold curve (*black dashed*) is denoted *F*. **a** Folded saddle geometry according to the singular reduced problem. The folded singularity resembles an ordinary saddle equilibrium with stable and unstable invariant manifolds (*red*). The trajectories (*blue*) follow these invariant manifolds moving away from the stable manifold and toward the unstable. **b** Within the desingularized reduced problem the dynamics on the repelling surface, $S^{r}$, are reversed due to the rescaling of time (desingularization). Trajectories may pass through the folded saddle with non-zero velocity traveling either of the invariant manifolds. These trajectories correspond to singular canards; the stable invariant manifold to the true canard and the unstable invariant manifold to the faux canard. **c** Folded saddle geometry in 3D. The true canard acts as a separatrix on the attracting surface, $S^{a}$. If a trajectory lands within the region enclosed by the true and faux canards, then it is bound away from the fold curve. However, if the trajectory lands within the region enclosed by the fold curve and true canard it travels toward the fold curve. Here, the trajectory “jumps off” due to a blow up in finite time of the desingularized reduced problem, where subsequent dynamics are dictated by the layer problem. The region within which trajectories necessarily ‘jump off’ the critical manifold is indicated (*purple shaded*)

**Fig. 6:**
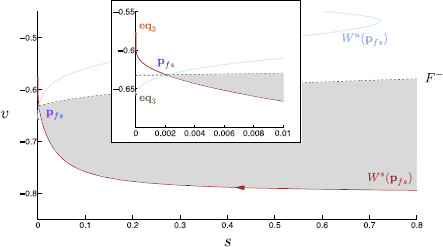
The desingularised reduced problem. The desingularized reduced problem, (15), near the lower fold is projected onto (v,s)-space. Here, $\tau_{s}=15$, although the basic structure shown here is common to all values of $\tau_{s}$ analyzed. The lower fold curve, $F^{-}$ (*gray dashed*), is indicated. The folded saddle, $p_{\mathrm{fs}}$ (*purple*), lies on the fold curve and gives rise to two invariant manifolds: a stable invariant manifold, $W^{s}(p_{\mathrm{fs}})$ (*red*), and an unstable invariant manifold, $W^{u}(p_{\mathrm{fs}})$ (*blue*). The dynamics in (v,s)-space above $F^{-}$ are reversed, and so the stable and unstable manifolds have reversed stability properties above $F^{-}$. *Arrows* indicate motion along the invariant manifolds. Each manifold terminates at a stable node equilibrium, within the reduced flow. The stable manifold terminates at ${\mathrm{eq}}_{2}$ on $S_{a}^{-}$ (*orange*) and the unstable manifold terminates at ${\mathrm{eq}}_{3}$ on $S_{r}^{-}$ (*green*). Note, if a singular trajectory lands onto the shaded region of $S_{0}$, it eventually undergoes a rebound spike. *Inset*: A magnification of the desingularized reduced problem near the folded saddle

We also find folded singularities on both the upper and lower folds of $S_{0}$. In contrast to the set of ordinary singularities, the position and stability properties of the folded singularities are highly dependent on $\tau_{s}$. Only singularities on the lower fold curve are expected to influence the dynamics significantly within the reduced problem as only this fold lies near a physiologically relevant attracting sheet of equilibria. For all values of $\tau_{s}$ here considered, there exists a single folded saddle, $p_{\mathrm{fs}}$, on the lower fold curve of $S_{0}$ in the physiologically relevant range, s∈[0,1]. The local geometry of a folded saddle singularity is illustrated within Fig. 5. Recall that we reverse the direction of the desingularized flow on the repelling middle branch of $S_{0}$ to obtain the correct reduced flow (Fig. 5b). We identify singular folded saddle canards as trajectories on $S_{0}$, which pass through the folded singularity, $p_{\mathrm{fs}}$, from the attracting critical manifold surface, $S_{a}^{-}$, and follow the repelling critical manifold surface, $S_{r}^{-}$, for a considerable amount of time. Here, we find two canard trajectories; the true canard, $\xi_{t}$, and the faux canard, $\xi_{f}$. The faux canard is the particular solution trajectory that lies initially on the repelling surface of the manifold, passes through the folded singularity and onto the attracting surface of the manifold (cf. Figs. 5 and 6). These canards correspond to the global invariant manifolds of the folded singularity, i.e., $\xi_{t}=W^{s}(p_{\mathrm{fs}})$ and $\xi_{f}=W^{u}(p_{\mathrm{fs}})$. A diagram of the reduced problem, around $F^{-}$, is given for $\tau_{s}=15$ in Fig. 6. The basic structure of the reduced problem, under variation of the parameter $\tau_{s}$, is stretched further along the fold curve in the positive *s*-direction for larger $\tau_{s}$ and contracts toward s=0 for smaller $\tau_{s}$ values. 

The folded saddle canard $W^{s}(p_{\mathrm{fs}})$ is a separatrix and effectively organizes the solution trajectories of the reduced problem. Depending on which side of $W^{s}(p_{\mathrm{fs}})$ the trajectory is initially, solution trajectories of (15) travel along $W^{s}(p_{\mathrm{fs}})$ and either meet the fold curve for $s>s_{\mathrm{fs}}$ or move toward, later traveling along, $W^{s}(p_{\mathrm{fs}})$. In the former case, the trajectory is no longer accurately approximated by the reduced problem at the fold due to a finite time blow up of system (15) and subsequent dynamics are dictated by the layer problem. In the latter case, the solution trajectory terminates at ${\mathrm{eq}}_{3}$, prevented by $W^{s}(p_{\mathrm{fs}})$ from approaching $F^{-}$. Compare Figs. 5 and 6.

## 5 Firing Threshold Manifolds and Dynamic Inhibition

Using information from the reduced and layer flows, we are able to give a singular limit prediction of post-inhibitory rebound spiking as observed in system (1). Recall that the critical manifold is given by the set of equilibria of the layer problem. Trajectories of the layer problem approach this set along so-called *fast fibres*. In this context, the critical manifold forms the set of *base points* of these fast fibers. Hence, base points allow for a connection between the flows of the layer and reduced problems. In particular, the relationship between the base point of the fast fibre through the initial condition due to inhibition and the singular canard solution determines whether a trajectory goes on to spike or not; see Fig. 9.

**Fig. 7:**
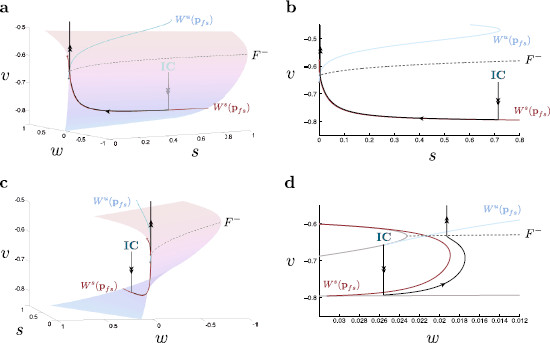
Singular global trajectories in (v,w,s)-space. The desingularized reduced problem is projected onto the critical manifold near the lower fold (*gray dashed*) for $\tau_{s}=15$. Singular solution trajectories from the layer problem, (7), and the reduced problem, (15), are concatenated to produce singular global trajectories (*black*). **a** From the initial condition (*IC*), the layer problem dictates that the singular trajectory falls onto $S_{a}^{-}$. Since the singular trajectory base point lies within the region bound between the canard separatrix and $F^{-}$, the reduced problem dictates that the trajectory evolves toward the fold curve. At the fold, due to a singular blow up of the reduced problem, the trajectory undergoes fast oscillations within the layer problem. This trajectory corresponds to a successful post-inhibitory rebound spike. **b** The corresponding system projected onto (v,s)-space. **c** The three-dimensional system from a different angle. Note the initial approach of the trajectory onto the critical manifold. **d** The corresponding system projected onto (v,w)-space. Note, this view provides a clear delineation between the singular canard and singular trajectories while the others do not. Hereafter, this projection is used when comparing canards and their respective trajectories

Singular global trajectories are constructed as continuous concatenations of reduced problem and layer problem solution trajectories. These concatenated dynamics are presented in (v,w,s)-space in Fig. 7. In modeling post-inhibitory rebound spiking, the state point is initially set at ${\mathrm{eq}}_{3}$. This stable node equilibrium simulates the resting membrane potential of the neuron. To model a pulse of inhibitory synaptic input, the trajectory undergoes an instantaneous shift in the *s*-direction such that $s_{0}=0.714$. Recall that this shift simulates a GABAergic IPSC within a network of neurons. From this point, the dynamics of the system take effect. This synaptic input necessarily translates the trajectory (instantaneously) to a position not on $S_{0}$ due to the shape of the manifold. Hence, the layer problem describes the initial motion from this point. Here, the trajectory rapidly approaches an attracting surface of the critical manifold, invariably $S_{a}^{-}$, along a fast fibre through this initial condition. This initial fast motion of the singular solution trajectory toward the manifold is best seen in Fig. 7c. 

**Fig. 8:**
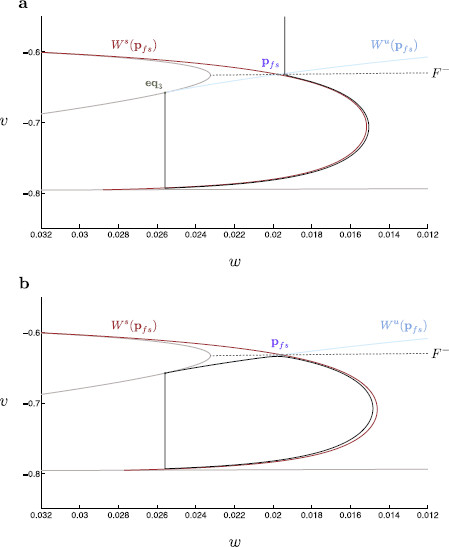
Singular limit spiking activity transitions. Singular solution trajectories from the layer problem, (7), and the reduced problem, (15), are concatenated to produce singular global trajectories (*black*). The singular limit predicts a range of $\tau_{s}$ values for which rebound spiking occurs; $\tau_{s}\in [5,24]$. The layer problem dictates that the trajectory has a base point on $S_{a}^{-}$ independent of $\tau_{s}$. Once on the manifold, the reduced problem dictates that the trajectory remains to one side of the canard separatrix. **a** The singular trajectory for $\tau_{s}=24$ in (v,w)-space. Since this trajectory lies to the right of the separatrix, it evolves in time toward the fold curve, $F^{-}$ (*gray dashed*), at which point the layer problem describes the onset of oscillatory behavior. This singular prediction corresponds to a successful rebound spike. **b** The singular trajectory for $\tau_{s}=25$ in (v,w)-space. This trajectory lies to the left of the separatrix and evolves in time toward ${\mathrm{eq}}_{3}$ (*green*). This singular prediction corresponds to an unsuccessful rebound spike. An animation of this figure under variation of $\tau_{s}$ is given within Additional file 1

The specific base point of the fast fibre through the initial condition acts then as an initial condition for the reduced system dynamics. This base point is independent of $\tau_{s}$ due to the lack of *s* dynamics within the layer problem. As determined above, there exists a general organizing structure within the reduced problem that determines the subsequent motion of a given trajectory. This structure, termed the canard separatrix, is the portion of the singular canard, $\xi_{t}$, which lies on $S_{a}^{-}$. The position of the separatrix varies according to the value of $\tau_{s}$ while the specific base point on $S_{0}$ remains constant. If the base point of the solution trajectory falls within the region on $S_{a}^{-}$ bound by $\xi_{t}$ and $F^{-}$, i.e., the shaded region in Figs. 5 and 6, then the trajectory evolves toward the fold curve at which point the dynamics of the trajectory are described by the layer problem. Here, the layer problem dictates that the trajectory undergoes a (fast) oscillation. We observe that the trajectory oscillates only once, during which the reduced problem dictates an average shift in the direction of positive *w*, and positive *s*. This shift causes the trajectory to “fall off” the oscillatory regime and return to $S_{a}^{-}$ on the other side of the canard separatrix. According to the reduced problem, the trajectory finally comes to rest at ${\mathrm{eq}}_{3}$. This singular global trajectory corresponds to a successful post-inhibitory rebound spike. If, however, the base point falls within the region on $S_{a}^{-}$ bound by $\xi_{t}$ and $\xi_{f}$ and characterized by smaller *s*-values, the solution trajectory is guided along the true and then the faux canard, coming to rest at ${\mathrm{eq}}_{3}$ without any fast oscillatory behavior. This singular global trajectory corresponds to a failure to spike after inhibition. The upper transition of spiking behavior is illustrated in Fig. 8. 

**Fig. 9:**
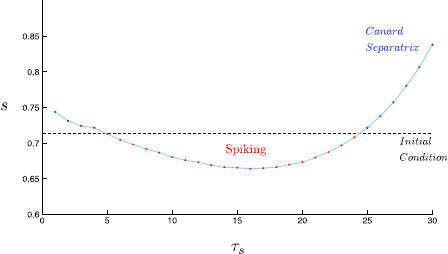
Singular limit prediction of spiking. Within the singular limit, the position of the base point determines whether a singular trajectory results in a rebound spike. We plot the *s*-value of the trajectory initial condition (*black dashed*), which coincides with the base point on the critical manifold. Note that the initial condition, and thus the base point is independent of $\tau_{s}$. For integer values of $\tau_{s}$, the *s*-value at which the corresponding canard separatrix crosses the *v*-value of the base point (*red/blue points*) is plotted. Thus, if an initial condition lies at a higher *s*-value than the corresponding canard separatrix (*red*), the trajectory falls onto the region of $S_{a}^{-}$, for which a trajectory rebound spikes. Otherwise, if the initial condition lies at a lower *s*-value than the corresponding canard separatrix (*blue*), the trajectory does not go on to spike. A linear interpolation shows the roughly parabolic shape, which gives rise to two rebound spiking transitions, and thus a single range of $\tau_{s}$ for which there exists rebound spiking. An animation of this figure under variation of $\tau_{s}$ is given within Additional file 1

*The Singular Limit Predicts a Window of Rebound Spiking with Increasing GABAa Inhibition* We obtain a singular limit prediction of post-inhibitory rebound spiking by simply identifying the position of the base point of the initial condition relative to the position of the singular canard under variation of $\tau_{s}$. Figure 9 shows that rebound spiking is predicted for $\tau_{s}\in [5,24]$. This is a good approximation to the range of rebound spiking within the full six-dimensional system; $\tau_{s}\in [8,21]$. An additional movie file illustrates this shifting of the folded-saddle canard and its effect on spiking behavior in the singular limit [see Additional file 1]. Looking at Fig. 9, the corresponding shift in the *s*-value falls within the order of the singular perturbation parameter *ε*, as expected by a singular perturbation analysis. 

### 5.1 Nonsingular Canards: The True Threshold Manifolds for Spiking Activity

An important result from GSPT is that a singular canard of folded saddle type perturbs to a nearby canard for the full model problem [21]. Consequently, the perturbed canard forms the true firing threshold manifold of the full system (1). In the following, we confirm numerically this firing threshold manifold by calculating the canard. We note here that due to numerical challenges in calculating canards for systems in $R^{n}$, n>3 with more than one fast variable, we consider a reduction of the six-dimensional modified propofol model. Resolving this issue is left as a point of focus for future work. By setting each of *m*, *h* and *n* to their respective steady-state values, we derive a three-dimensional reduction of our original system. This reduction is valid as we have determined that *m*, *h* and *n* evolve on a fast time-scale; this system simply approximating this fact by having these processes act instantaneously. Within this reduced system the range of $\tau_{s}$ values for which spiking occurs is similar to the original with $\tau_{s}\in [9,21]$; however, the spiking mechanism is partially disabled and the trajectory is unable to fully reset. At least 2 fast variables are necessary to provide the necessary repolarization mechanism. We note that this does not affect sub or perithreshold dynamics, and consequently does not affect the onset of spiking. Hence, a model reduction to 1 fast variable locally near the fold is justified.

Using the continuation package AUTO [7], and closely following the techniques outlined in [5], the nonsingular canard, $\Xi_{t}$, is found for $\tau_{s}=15$ and is then continued in $\tau_{s}$. The nonsingular canards of the modified propofol model lie at the intersection of the attracting and repelling slow manifolds, $S_{\varepsilon }^{a}$ and $S_{\varepsilon }^{r}$, respectively. These manifolds correspond to $S_{a}^{-}$ and $S_{r}^{-}$, respectively, for ε>0. The attracting and repelling manifolds of the perturbed system are calculated using the homotopy continuation of solution trajectories to a suitable boundary value problem. Here, we make use of the normal hyperbolicity of the critical manifold $S_{0}$. Namely, for small ε>0, the slow manifolds $S_{\varepsilon }^{a}$ and $S_{\varepsilon }^{r}$ are smooth perturbations of the critical manifold $S_{0}$, away from the fold curve *F* where normal hyperbolicity is lost. A detailed description of slow invariant manifold calculations and canard detection and continuation is provided for the self-coupled FitzHugh–Nagumo system within the AUTO manual, demo **fnc**.

**Fig. 10:**
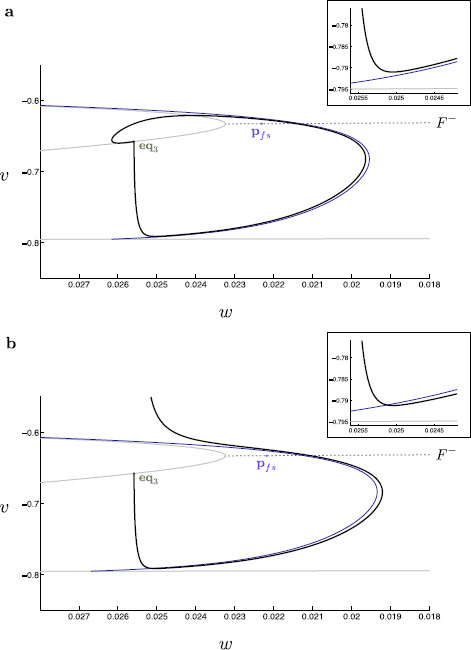
Nonsingular canards and global trajectories. The true nonsingular canard (*blue*) for the three-dimensional modified propofol model is located for $\tau_{s}=15$. This canard is then continued in $\tau_{s}$ using a suitable boundary condition problem. We thus obtain the true canard for any value of $\tau_{s}$. This true canard is compared to the corresponding trajectory (*black*) of the three-dimensional modified propofol model. Here, we see that the true nonsingular canard correctly bounds the trajectories into spiking and quiescent regimes. The folded singularity (*purple*) and ${\mathrm{eq}}_{3}$ (*green*) are indicated. **a** The nonsingular trajectory and associated canard for $\tau_{s}=8$ in (v,w)-space. **b** The nonsingular trajectory and associated canard for $\tau_{s}=9$ in (v,w)-space

Within Fig. 10, we find that the nonsingular canard accurately forms the boundary of the spiking and nonspiking behavior within the three-dimensional modified propofol model. We thus confirm that the canard does in fact form the threshold manifold of spiking activity. This nonsingular analysis confirms our geometric understanding of the singular limit prediction on both interval boundaries. 

## 6 Firing Threshold Manifolds and the Classical Step Protocol

**Fig. 11:**
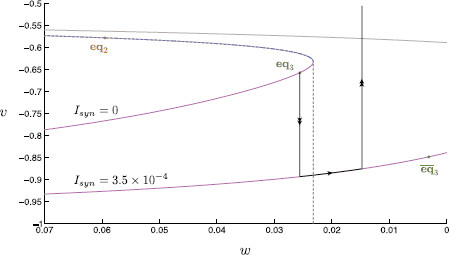
Singular limit analysis of step inhibition. A classical current step protocol is applied to the dimensionless modified propofol model. A geometric singular perturbation analysis is then performed on this system. The synaptic current is held at $I_{\mathrm{syn}}=3.5\times 10^{-4}$ to simulate inhibition. We compare the critical manifold for each value of $I_{\mathrm{syn}}$. On each manifold, the lower branch (*magenta*) is linearly stable, the upper branch (*gray*) linearly unstable. The stable node equilibria (*green*), ${\mathrm{eq}}_{3}$ and ${\bar{\mathrm{eq}}}_{3}$, and saddle equilibrium (*orange*), ${\mathrm{eq}}_{3}$ are indicated. Initially, the system starts at rest on the node equilibrium of the $I_{\mathrm{syn}}=0$ critical manifold. At the onset of inhibition, the critical manifold is shifted in the direction of negative *w*. In the singular limit, the layer problem dictates that the trajectory falls to the lower branch of the critical manifold. Once on the manifold, the reduced problem dictates that the trajectory slowly approaches ${\bar{\mathrm{eq}}}_{3}$. Once the inhibitory current is removed, the manifold shifts back to its original position, at which point the layer problem determines that the trajectory shoots upward in the direction of positive *v*. In the singular limit, the threshold manifold (*blue dashed*) is the concatenation of the middle branch of the critical manifold with the fast fibre through the lower fold of the critical manifold. If the singular limit trajectory passes this manifold, the singular limit predicts a spike event

We have thus far shown that it is necessary to consider the effects of dynamic inhibition in order to understand the cessation of spiking for large $\tau_{s}$. Here, we analyze the current step protocol and seek to understand why only a single transition of activity can be found. We again use singular perturbation analysis to identify the spiking threshold manifold. In the singular limit, this manifold is given by the concatenation of the middle branch of the one-dimensional critical manifold and the fast fibre through a fold point when $I_{\mathrm{app}}=0$ (see Fig. 11). 

### 6.1 Singular Perturbation Analysis

In a singular perturbation analysis similar to that above, we consider four fast variables, (v,m,h,n), and a single slow variable, *w*. Note that *s* dynamics are no longer considered in a current step protocol; $I_{\mathrm{syn}}$, and thus dependence on *s*, is here replaced by a constant value. In the singular limit, the layer problem defines a one-dimensional cubic-shaped critical manifold in (v,w)-space. As per the above analysis, only the lower branch of the critical manifold is an attracting branch; the upper two branches being unstable. While the shape and stability properties of the critical manifold are independent of the value of $I_{\mathrm{syn}}$, the position of the critical manifold is shifted in the direction of negative *w* as the synaptic current is set to $I_{\mathrm{syn}}=3.5\times 10^{-4}$ during inhibition (otherwise $I_{\mathrm{syn}}=0$; see Fig. 11). Note that here the critical manifold for $I_{\mathrm{syn}}=0$ is precisely the section defined by {s=0} through the critical manifold derived above, i.e., the critical manifold for $I_{\mathrm{syn}}=0$ is here given by $S_{0}\cap \{s=0\}$. Once on the critical manifold, dynamics are described by the reduced problem; here formulated as (10) with $s^{\prime }=0$ and $I_{\mathrm{syn}}$ adjusted as per the step protocol. The reduced problem reveals, as before, the existence of three stable node equilibria, one on each branch of the critical manifold. We label the equilibria as before for $I_{\mathrm{syn}}=0$, whereas the equilibria on the shifted critical manifold, i.e., for $I_{\mathrm{syn}}=3.5\times 10^{-4}$, are denoted ${\bar{\mathrm{eq}}}_{i}$, i=1,2,3.

Initially, the state point is held at rest at ${\mathrm{eq}}_{3}$ with $I_{\mathrm{syn}}=0$. At the onset of synaptic inhibition the critical manifold shifts, and so defines a new stable equilibrium, ${\bar{\mathrm{eq}}}_{3}$. In the singular limit (Fig. 11), the trajectory falls instantaneously, traveling along a vertical fast fibre, onto the stable branch of the shifted critical manifold. This base point is thus a projection of ${\mathrm{eq}}_{3}$ onto the shifted critical manifold. The singular trajectory is then described by the reduced problem, slowly moving along this lower branch toward ${\bar{\mathrm{eq}}}_{3}$. Once synaptic inhibition is removed, the critical manifold shifts back to its original position. At this point, the layer problem dictates a rapid vertical ascent; the trajectory no longer remains on the critical manifold. The subsequent dynamics are thus dependent on the length of the applied constant synaptic inhibition. If the duration of inhibition is relatively short, at the removal of inhibition, the trajectory simply returns to the now overhead lower branch of the critical manifold. This corresponds to an unsuccessful post-inhibitory rebound spike. If; however, the duration of inhibition allows the trajectory time enough to sufficiently approach ${\bar{\mathrm{eq}}}_{3}$ then, at the removal of inhibition, the trajectory undergoes a spike in *v*. This behavior corresponds to a successful post-inhibitory rebound spike. The associated threshold manifold is given as the concatenation of the middle branch of the critical manifold and the fast fiber through the lower fold point, shown in Fig. 11.

#### 6.1.1 The Singular Limit Predicts Only One Spiking Transition with Increasing Duration of Inhibition

We here determine that the singular limit predicts the minimum duration of inhibition such that a trajectory is still able to spike is 6 ms. Since the layer problem acts instantaneously, this value is determined solely according to the dynamics within the reduced problem. We simulate the singular reduced problem along the lower branch of the critical manifold, noting the time at which the singular trajectory passes the *w*-value of the lower fold. Note here we use the singular problem to avoid the distortion of time within the desingularized problem due to a position dependent rescaling of time in the process of desingularization.

### 6.2 Nonsingular Firing Threshold Manifold

**Fig. 12:**
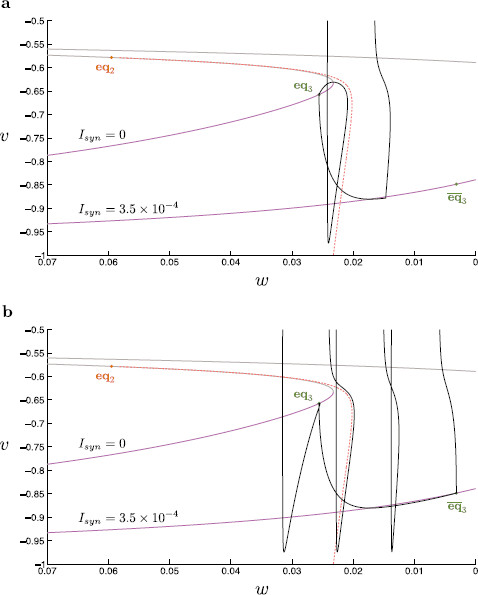
Non-singular analysis of step inhibition. A classical current step protocol is applied to the dimensionless modified propofol model for 40 ms, and 250 ms. We compare the critical manifolds (*v*-nullclines) for $I_{\mathrm{syn}}=0$ and $I_{\mathrm{syn}}=3.5\times 10^{-4}$. On each manifold, the lower branch (*magenta*) is linearly stable, the upper branch (*gray*) linearly unstable. The corresponding stable node equilibria (*green*), ${\mathrm{eq}}_{3}$ and ${\bar{\mathrm{eq}}}_{3}$, and saddle equilibrium (*orange*), ${\mathrm{eq}}_{2}$, are indicated. Initially, the system starts at rest at the node equilibrium of the $I_{\mathrm{syn}}=0$ critical manifold. At the onset of inhibition, the manifold is shifted in the direction of negative *w*. The trajectory falls to the lower branch of the shifted manifold, slowly approaching ${\bar{\mathrm{eq}}}_{3}$. Once the inhibitory current is removed, the critical manifold shifts back to its original position, at which point the trajectory rapidly shoots in the direction of positive *v*. The threshold manifold for trajectory spiking is indicated (*orange dashed*). **a** If the trajectory passes this separatrix, when released from inhibition, the system spikes. The trajectory is reset with a net shift in the direction of positive *w*. Upon resetting, the trajectory lies to the left of the separatrix and falls within the basin of attraction of the stable node equilibrium. **b** As the length of inhibition is increased, the trajectory evolves closer toward the shifted equilibrium position. Here, the trajectory undergoes three spikes before being reset to the left of the separatrix and finally coming to rest. Note the system used here to calculate the threshold manifold makes use of the reduction $\tilde{x}={\tilde{x}}_{\infty }(v)$, for x=m,h,n, in order to allow calculation along the repelling middle branch. Locally (for sub and perithreshold regimes) this reduced system well approximates the modified propofol model, despite being unable to repolarize after a spiking event

Here, as expected, the singular limit accurately mimics the behavior of the nonsingular system (compare Figs. 11 and 12). As per the singular limit prediction, the duration of inhibition determines whether or not a given trajectory spikes in the nonsingular system (Fig. 12). We observe that for short step protocols, the corresponding trajectory makes only a small excursion down toward the shifted critical manifold without reaching it. Once synaptic inhibition is released the trajectory returns to ${\mathrm{eq}}_{3}$ shortly after. In a voltage time-trace, this behavior manifests in a short dip in *v* before a return to the resting membrane potential (Fig. 3a). As the step protocol is lengthened, the trajectory reaches the shifted critical manifold, afterward slowly moving toward ${\bar{\mathrm{eq}}}_{3}$ (Fig. 12a). For synaptic inhibition lasting longer than 14 ms, when inhibition is removed, the local attractor is found within a fast oscillatory regime. Here, we observe a small dip in *v* followed by a spike within the voltage time-trace (Fig. 3b). 

#### 6.2.1 A Canard Solution Forms the Nonsingular Separatrix Between Spiking and Nonspiking Solutions

As per the analysis of dynamic inhibition above, we again find a geometric object which demarcates the boundaries of trajectories with different spiking behavior. Within the nonsingular system the perturbed separatrix is located using a shooting method, in backward time, from the stable node ${\mathrm{eq}}_{2}$. As before, the propofol model used to calculate this separatrix requires the reduction $\tilde{x}={\tilde{x}}_{\infty }(v)$, for x=m,h,n, in order to allow calculation along an unstable manifold. Here, we see that the nonsingular separatrix forms the boundary of spiking and nonspiking behavior (compare Figs. 12a and 12b). We note here that the threshold manifold is a canard, as per the dynamic inhibition analysis. Accordingly, we find that trajectories, which begin exponentially close to this structure follow it for a significant amount of time, even onto a repelling portion of the associated critical manifold, such as that in Fig. 12b near the fold point. The deviation of the perturbed threshold manifold compared with that of the singular analysis near the critical manifold lower fold is explained by a fold analysis within singular perturbation theory (compare Figs. 11 and 12). Here, we expect, and indeed find, that the nonsingular manifold perturbs a distance which is $O(\varepsilon^{2/3})$ from the fold [16,22]. Hence, we have identified the non-singular threshold manifold for the current step protocol. 

#### 6.2.2 Explanation of a Finite Number of Rebound Spikes as Inhibition Time Is Increased

As the duration of the step protocol increases, the number of rebound spikes increases steadily to a maximum of three spikes (compare Figs. 3d and 12b). This observation is explained by a limited reset in the direction of *w* during each rebound spike. After a single spike, as seen in Fig. 12b, the corresponding trajectory remains to the right of the threshold manifold. Three consecutive rebound spikes are required before the trajectory returns to an excitable resting state at ${\mathrm{eq}}_{3}$, having moved past the threshold manifold. At this point, we see that further prolonging the duration of synaptic inhibition has no additional effect. Once the trajectory is sufficiently close to ${\bar{\mathrm{eq}}}_{3}$ during synaptic inhibition, regardless of the actual duration of inhibition, the subsequent dynamics remain unchanged. This results in a maximum number of rebound spikes. This maximum spike number is encoded within the time-scale separation between the fast and slow dynamics, i.e., within *ε*. For fixed *ε*, the maximum spike number is set according to the average shift in the slow dynamics per (fast) spike event. As ε→0, we observe that the maximum spike number increases as the reset in *w* reduces per spike (work not shown). The singular limit picture confirms this finding, here showing no net shift in *w* per spike for ε=0.

### 6.3 The Folded Saddle Canards of the Dynamic Protocol Converge Toward the Spiking Threshold Manifold of the Current Step Protocol as $\tau_{s}\to 0$

**Fig. 13:**
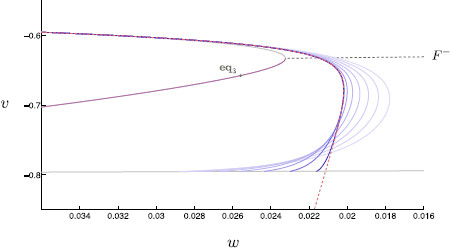
Firing threshold manifolds: dynamic vs. static inhibition. Firing threshold manifolds of dynamic inhibition and static inhibition are here plotted together. Recall the firing threshold manifolds for dynamic inhibition are the nonsingular canards, here shown for odd values of $\tau_{s}$ between 1–15 (*solid purple*). The firing threshold manifold for static inhibition is the stable manifold of ${\mathrm{eq}}_{2}$ (orange dashed) shown earlier in Fig. 12. For decreasing values of $\tau_{s}$ (*darker shades of purple*), the nonsingular canards more closely resemble the static inhibition threshold manifold. Note each system (dynamic or static inhibition) used to calculate a threshold manifold required the reduction $\tilde{x}={\tilde{x}}_{\infty }(v)$, for x=m,h,n, to allow calculation along repelling manifolds

In the modified propofol model, we are able to explain both spiking transitions through identification of the firing threshold manifold in a 4-fast/2-slow time-scale separation setting—the threshold manifold being the folded saddle canard. Using the software package AUTO, we continue this folded saddle canard toward small $\tau_{s}$ values. Figure 13 shows the firing threshold of the modified propofol model for $1\leq \tau_{s}\leq 15$ (solid purple) as well as the firing threshold manifold of the current step protocol (dashed orange). Clearly, the folded saddle canards converge toward the firing threshold manifold of the current step protocol as $\tau_{s}\to 0$. We note that as $\tau_{s}\to 0$ we consider *s* a fast variable within a GSPT analysis. Given that the *s* dynamics are decoupled from the rest of the system, we can easily identify the stable equilibrium state; s=0. Thus, in the limit as $\tau_{s}\to 0$, the system dynamics and, therefore, the firing threshold manifold more closely represents that of a system where the *s*-dynamics rapidly equilibrate to s=0; i.e., the step protocol where $I_{\mathrm{syn}}=0$. Hence, we show the intimate relationship between the two identified distinct firing threshold manifolds in the modified propofol model, the folded saddle canard, and the stable manifold of the saddle equilibrium. 

## 7 Comparison of the Original and Modified Propofol Models

The propofol network model presented here is a modified form of the propofol neuron network model in [18]. The propofol model is the same 6–dimensional system of equations (Eqs. (1) and (2)) as the modified propofol model but with two parameter changes. The modified propofol model is phenomenologically similar to the original propofol model, which shows rebound spiking only for values of $\tau_{s}$ between 8 and 48 ms (compared with the modified model: 8–21 ms) and was also examined using geometric singular perturbation theory [18]. In particular, a canard of folded saddle type was identified as a firing threshold manifold. In Sects. 7.1–7.3, we detail our reasons for modifying the original propofol model.

### 7.1 Modifying the Propofol Model: Increasing the Time-Scale Separation Allows for a more Accurate Singular Limit Trajectory Approximation

Both models, the original and the modified propofol model, have a global stable equilibrium that plays the role of the resting membrane potential. We note that this stable equilibrium (*v*-value of −63.6 mV) of the original propofol model lies quite close to the fold curve, $F^{-}$. Additionally, we observe that the fold-curve $F^{-}$ has an almost constant *v*-value (v≈−63 mV along $F^{-}$). Given the fairly uniform structure of the critical manifold $S_{0}$ in the direction of *s* near $F^{-}$, the position of the stable equilibrium ensures a proximity of the post-inhibitory initial condition and thus of the post-inhibitory dynamics to $F^{-}$.

**Fig. 14:**
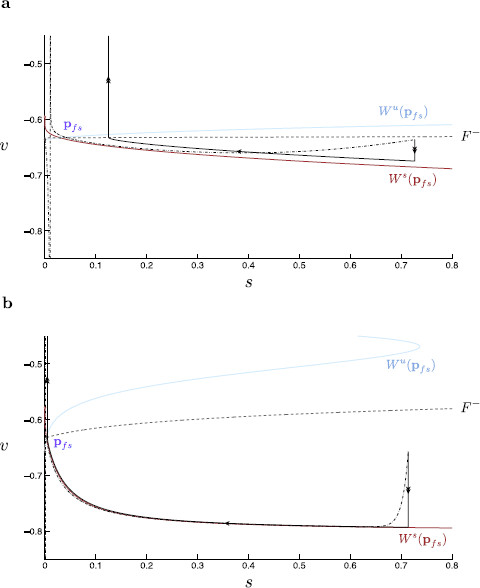
Singular and nonsingular trajectories of the original and modified propofol model. Singular global trajectories (*black*, *solid*) are here overlaid with their corresponding nonsingular propofol model trajectories (*black*, *dashed*). Here, we compare the original and modified propofol model solution trajectories and singular limit predictions for $\tau_{s}=20\text{ ms}$ (spiking occurs in both models for this value). **a** Within the original propofol model, solution trajectories are kept in close proximity to the lower fold curve. Near the fold, normal hyperbolicity is lost and the time-scale separation between fast and slow variables breaks down. Hence, the singular trajectory does not accurately describe the fast approach onto the critical manifold. This results in a substantial deviation of solution trajectories from the singular limit prediction of subsequent slow dynamics; note, for example, the discrepancy between the *s*-values at which the non-singular and singular trajectories spike. **b** The modified propofol model solution trajectories are no longer constrained near the fold curve, and thus the time-scale separation is preserved. This results in a more accurate singular limit prediction of nonsingular trajectories

This proximity to a fold structure is known to affect the time-scale separation of variables within a singularly perturbed problem. More specifically, near normally hyperbolic equilibria, fast and slow dynamics within a singularly perturbed system evolve as O(1) and O(ε), respectively. Within a neighborhood of a fold structure, at which normal hyperbolicity is lost, the original fast/slow splitting does not persist and the fast and slow dynamics evolve predominantly on an intermediate time-scale $O(\varepsilon^{1/3})$ and a slow time-scale O(ε)[22]. This explains why we find an appreciable deviation between the singular and nonsingular trajectories in the original propofol model (Fig. 14a). 

Thus, we propose the modified propofol model of post-inhibitory rebound spiking. This modified propofol model retains the basic phenomenological and geometric features of the propofol model while the position of the stable equilibrium has been moved away from the fold curves. This modification allows for a more marked separation of time-scales during the early dynamics, and thus describes a system more accurately approximated by a singular limit prediction (Fig. 14b).

To formulate the modified propofol model the steady-state function $w_{\infty }(v)$ is shifted by 3 mV in the direction of negative *v*. This results in a lower global stable equilibrium point (*v*-value of −65.8 mV), and thus a lower resting membrane potential. However, this modification also makes it more difficult to generate a rebound spike. In order to counter this effect, the value of the maximal synaptic conductance $g_{i}$ is increased so as to effectively increase the strength of synaptic inhibition. Here, we set $g_{i}$ to 4 mS, i.e., ${\bar{g}}_{i}=0.04$.

As previously noted, the behavior of the modified propofol model is similar to the original propofol model in that post-inhibitory rebound spiking occurs only for intermediate values of $\tau_{s}$ (Fig. 2). Thus, by our modifications, we have not lost the rebound-spiking “window” and we have gained a more accurate geometrical representation of nonsingular trajectories by their singular limit. However, even more importantly, the modified model allows us to use the same fast/slow decomposition to predict the spiking transition at both interval boundaries ($\tau_{s}$ small and $\tau_{s}$ large). This was not possible in the original propofol model. We describe this in more detail next.

### 7.2 Discrepancies Between the Singular and Nonsingular Trajectories in the Propofol Model

Although the singular limit of the original propofol model provides a good approximation for cessation of spiking when $\tau_{s}$ is large (52 ms for the singular limit versus 48 ms for nonsingular trajectories), when we compare singular global trajectories with the nonsingular trajectories of the propofol model, we find that the geometry of the singular limit trajectories does not accurately predict the geometry of the nonsingular trajectories (Fig. 14). In particular, the early dynamics of the singular limit trajectories do not accurately mimic those of the nonsingular trajectories using the proposed slow/fast splitting. As mentioned before, this poor predictive power of geometric singular perturbation theory can be explained by the proximity of the trajectory near the fold $F^{-}$, throughout its evolution. The time-scale splitting of the “fast” (v,m,h,n) and “slow” (w,s) variables away from the fold $F^{-}$ does not hold anymore in a neighborhood of the fold $F^{-}$. Without this time-scale splitting, and thus the identification of a singularly perturbed problem, regular GSPT analysis does not yield a reliable prediction of system dynamics.

We thus conclude that while the singular limit analysis still provides a good prediction for the large $\tau_{s}$ spiking transition in the original propofol model, the particular geometry of this system distorts the time-scale separation, and thus requires an alternative approach of geometric singular perturbation theory using a blow-up analysis along the fold-curve [22] followed by a blow-up of the folded saddle singularity [21,24]. This two-step approach is left for future work. 

### 7.3 In the Original Propofol Model, Considering *s* Slow or Fast Does not Explain the Spiking Transition when $\tau_{s}$ Is Small

In the original propofol model, considering *s* as a slow variable cannot predict the spiking transition when $\tau_{s}$ is small; see Fig. 10 in [18]. Similarly, considering *s* as a fast variable cannot predict the spiking transition when $\tau_{s}$ is small either; see explanation on p. 13 in [18]. Hence, using geometric singular perturbation theory in the context of either a 4-fast/2-slow time-scales or a 5-fast/1-slow time-scales separation does not predict the transition between spiking and nonspiking when $\tau_{s}$ is small in the original propofol model. The answer to this “riddle” about the appropriate dynamics of *s* for small $\tau_{s}$ in the original propofol model lies in an intermediate time-scale of order $O(\varepsilon^{\alpha })$, 0<α<1. Such an intermediate time-scale reveals itself when using a (cylindrical) blow-up analysis of the fold, $F^{-}$[22,24]. As mentioned above, we leave this blow-up analysis of the original propofol model for future work. 

### 7.4 Comparison of Geometric Structures in the Original and Modified Propofol Model when $\tau_{s}$ Is Small

In [18], the authors use a 5-fast/1-slow time-scale separation to examine the geometry of the spiking transition when $\tau_{s}$ is small. Interestingly, this results in the same resting state ($I_{\mathrm{syn}}=0$) singular limit geometry as the 4-fast/1-slow time-scale problem used here to analyze the current step protocol (Fig. 12). This makes sense because considering *s* fast allows the manifold {s=0} to be reached on a fast time-scale, and thus the system reduces to the same 4-fast/1-slow time-scale problem used here. As shown in [18], when *s* is considered one of the fast variables, as in the original propofol model, the stable manifold of ${\mathrm{eq}}_{2}$ in Fig. 12 approximates well the threshold for spiking. Recall that this is shown in Fig. 13, i.e., as $\tau_{s}\to 0$ the canard separatrix is well approximated by the firing threshold manifold of the current step protocol. Hence, by changing parameters to allow the construction via geometric singular perturbation theory of canards, this paper clarifies the underlying geometry of the original propofol model.

## 8 Concluding Remarks

An important feature of most physiological systems is that they evolve on multiple time-scales. The theory of differentiable dynamical systems for two time-scales (slow/fast) has a successful history in explaining a wide range of physiological behavior such as electrical spiking and bursting in neurons [13]. Complex pattern generation in such slow/fast systems is almost exclusively related to loss of normal hyperbolicity of invariant critical manifolds, which is associated with bifurcation sets in the fast subsystem. In particular, folded critical manifolds are ubiquitous in such systems (see, e.g., Fig. 4). Canard theory deals exactly with these slow/fast time-scales systems where loss of normal hyperbolicity occurs, and its theory is applicable to problems with arbitrary dimensions [2,3,8,15,16,21,23,24]. A recent success story of canard theory is that it provides an explanation for mixed-mode oscillations (MMOs), a frequently observed mix of small and large amplitude oscillation patterns in slow/fast time-scale physiological models. Canards of folded node and folded saddle-node type play a key role in explaining these patterns. The interested reader is referred to the current review on MMOs [5], and the extensive reference list to applications therein. 

Canard theory provides also a new direction for understanding transient dynamics of biological systems that have multiple time-scales. The propofol model studied here is a prime example. We demonstrate here the use of canard theory to explain the dynamics of rebound spiking for a specific range of propofol doses. In the context of neuronal excitability, we identify canards of folded saddle type as firing threshold manifolds. It is remarkable that dynamic information such as the temporal evolution of an external drive (GABAergic inhibition in this study) is encoded in the location of an invariant manifold—the canard. It is the variable positioning of the canard separatrix that explains the observed rebound spiking for a specific range of propofol doses (Fig. 9). The same role of folded saddle canards as firing threshold manifolds was recently identified in a class of Morris–Lecar/FitzHugh–Nagumo type models with dynamic external drive [25]. Since mathematical models of physiological phenomena (both neuronal and nonneuronal) frequently show abrupt transitions in behavior and have dynamics which are encoded by multiple time-scales, the methods of GPST and canard theory are likely applicable to a much broader range of problems within the biological sciences. 

## Appendix:  Steady State and Time-Scale Functions

The formulations for the steady state functions $\left(m_{\infty }(V),h_{\infty }(V),n_{\infty }(V)\right)$ and time-scale functions $\left(\tau_{m}(V),\tau_{h}(V),\tau_{n}(V)\right)$ are taken from [18]. The steady state function and time-scale function for *w* are here modified. These functions take the respective forms

(18) $$\begin{array}{rl} x_{\infty }(V) & =\frac{\alpha_{x}(V)}{\alpha_{x}(V)+\beta_{x}(V)},\\ \tau_{x}(V) & =\frac{\alpha_{x}(V)}{\alpha_{x}(V)+\beta_{x}(V)} \end{array}$$

for x=m,h,n,w. The constituent functions $\alpha_{x}(V)$ and $\beta_{x}(V)$ are given by

(19) $$\begin{array}{rcl} \alpha_{m}(V) & = & \frac{0.32(V+54)}{1-\exp[-(V+54)/4]},\\ \beta_{m}(V) & = & \frac{-0.28(V+27)}{1-\exp[(V+27)/5]},\\ \alpha_{h}(V) & = & 0.128\exp\left[-(V+50)/18\right],\\ \beta_{h}(V) & = & \frac{4}{1+\exp[-(V+27)/5]},\\ \alpha_{n}(V) & = & \frac{0.032(V+52)}{1-\exp[-(V+52)/4]},\\ \beta_{n}(V) & = & 0.5\exp\left[-(V+57)/40\right],\\ \alpha_{w}(V) & = & \frac{3.209\cdot 10^{-4}(V+33)}{1-\exp[-(V+33)/9]},\\ \beta_{w}(V) & = & \frac{3.209\cdot 10^{-4}(V+33)}{1-\exp[(V+33)/9]}. \end{array}$$

**Fig. 15:**
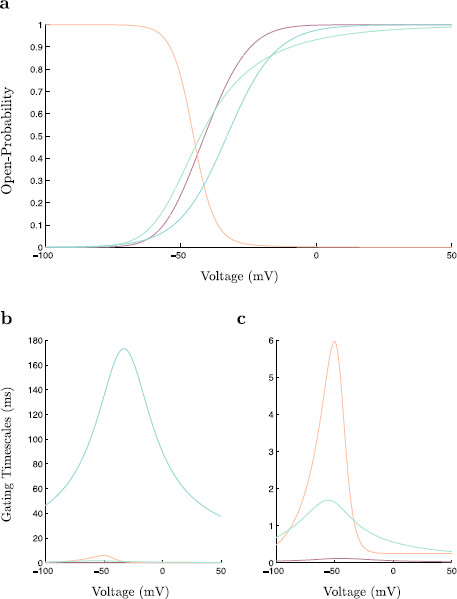
Gating variable steady-state and time-scale functions. **a** The four gating variable steady-state functions, $m_{\infty }(V)$ (*red*), $h_{\infty }(V)$ (*orange*), $n_{\infty }(V)$ (*green*), and $w_{\infty }(V)$ (*blue*). These functions give the opening probability of their respective ion channels for a given value of the membrane voltage, *V*. **b** The four gating variable time-scale functions, $\tau_{m}(V)$ (*red*), $\tau_{h}(V)$ (*orange*), $\tau_{n}(V)$ (*green*), and $\tau_{w}(V)$ (*blue*). These functions describe the rough time-scales on which each gating variable evolves for a given value of the membrane voltage. **c** A closer look at panel **b** reveals that $\tau_{m}(V)$, $\tau_{h}(V)$ and $\tau_{n}(V)$ evolve on much smaller time-scales than $\tau_{w}(V)$

**Fig. 16:**
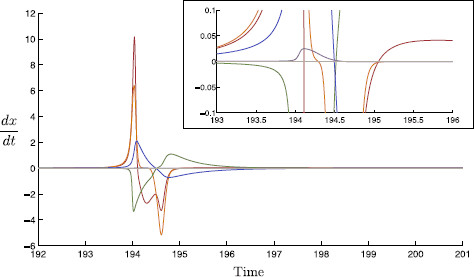
Variable time-scales. The time derivatives of each variable are plotted over a spike event; *v* (*red*), *m* (*orange*), *h* (*green*), *n* (*blue*), *w* (*purple*), and *s* (*gray*). We find that there is a clear order of magnitude difference between the time derivatives of the variables *v*, *m*, *h*, *n*, and *w*, *s*. *Inset*: A magnification of the derivative time-traces; here the relative magnitudes of $\frac{dw}{dt}$ and $\frac{ds}{dt}$ can be seen

The gating variable steady-state and time-scale functions are plotted in Fig. 15. The time derivative of each variable over the course of a single spike is given in Fig. 16. This figure indicates the actual time-scale separation and is used, in part, to justify a time-scale splitting as detailed in Sect. 3. 

## Electronic Supplementary Material

## Competing Interests

The authors declare that they have no competing interests.

## Authors’ Contributions

JM, MM, NC, and MW conceived of the analysis and of the overall goals of the project. JM carried out the numerical analysis and wrote the manuscript with assistance from MM, NC, and MW. All authors read and approved the final manuscript.

## Supplementary Material

Additional file 1 Here we present Fig. 8 and 9 in animated form. As tauS varies, the folded-saddle canard is shifted. This canard forms the firing threshold manifold and so the excitability of the cell is thus altered. The singular limit predicts a range of $\tau_{s}$ values for which rebound spiking occurs; $\tau_{s}\in [5,24]$. **a** Singular limit (i.e., ε=0) solution trajectories. The singular canard (*red*) and singular faux canard (*blue*) organize trajectories and govern excitability. The singular canard forms the firing threshold manifold. **b** Singular limit prediction of spiking. The base point remains independent of $\tau_{s}$ while the position of the singular canard varies with $\tau_{s}$. This alters excitability and gives rise to a window of $\tau_{s}$ values for which spiking occurs (MOV 264 kB) Click here for file
